# Nitric Acid for Whooping Cough

**Published:** 1878-06

**Authors:** 


Nitric Acid for Whooping-Cough.—

| | |
|---|---|
| ℞.—Nitric acid, dil., . . . . | 12 drachms. |
| Comp. tincture of cardamons, . | 3 drachms. |
| Syrup, . . . . . . | 2½ ounces. |
| Water, . . . . . . . | 1 ounce. |

Mix. One to two teaspoonfuls every two hours, according to age of child.

This is said by Sir G. D. Gibb to be a specific, and will cure in from two days to two weeks.—*Medical Brief.*

